# The experiences of prepregnancy care for women with type 2 diabetes mellitus: a meta-synthesis

**DOI:** 10.2147/IJWH.S115955

**Published:** 2016-12-08

**Authors:** Rita Forde, Evridiki E Patelarou, Angus Forbes

**Affiliations:** Department of Adult Nursing, Florence Nightingale Faculty of Nursing and Midwifery, King’s College London, London, UK

**Keywords:** systematic literature, pre-conception counseling, women’s health, contraception, meta-ethnography, patient education, lived-experience

## Abstract

**Background:**

Diabetes is one of the most common medical conditions affecting pregnancy and is associated with a number of adverse fetal, infant, and maternal outcomes. These adverse outcomes can be avoided or minimized with appropriate prepregnancy care (PPC). However, the uptake of PPC is limited in women with type 2 diabetes mellitus (T2DM). The reasons for poor uptake are multifactorial, reflecting both women’s understanding of pregnancy risks, and limitations in care delivery.

**Methods:**

A systematic literature review with meta-synthesis was undertaken to identify qualitative studies exploring experiences of PPC for women with T2DM incorporating the views of women with T2DM and health care professionals (HCPs). Identified studies included were synthesized in a meta-ethnography to develop an understanding of the elements contributing to the uptake of PPC among women with T2DM.

**Results:**

The systematic review identified seven studies yielding data from 28 women with T2DM and 83 HCPs. The following six third-order constructs were identified from the synthesis: understanding PPC, emotive catalysts, beliefs about reproduction among women with T2DM, relationships and social factors, HCP behaviors and perspectives, and health care system factors. These constructs were used to develop a multifactorial model expressing the interactive issues that shape the reproductive health-seeking behaviors of women with T2DM to identify potential areas for intervention.

**Conclusion:**

The uptake of PPC among women with T2DM seems to be informed by their personal orientation to their reproductive needs, their interactions with HCPs, and system-level influences. Future interventions to enhance PPC uptake need to address these underlying issues.

## Introduction

Diabetes is one of the most common medical conditions affecting pregnancy^1^ and is associated with adverse fetal, infant, and maternal outcomes.^2^ Many of these adverse outcomes can be avoided or minimized with appropriate care prior to and during pregnancy.^3^,^4^ Prepregnancy care (PPC) is particularly important as fetal development commences in the first trimester of pregnancy, before many women are aware of their pregnancy. Hence, current National Institute for Health and Care Excellence guidance stipulates that all women with diabetes considering pregnancy should receive PPC to normalize glucose levels, discontinue teratogenic therapies, and introduce high-dose folic acid.^5^

Traditionally, the focus on PPC has been on women with type 1 diabetes mellitus (T1DM). However, with increasing obesity levels, the prevalence of type 2 diabetes mellitus (T2DM) is rising among women of childbearing age^6^,^7^ and is particularly high in women of African or Asian origin.^8^ The recent UK National Pregnancy in Diabetes (NPID) audit showed that women with T2DM now constitute 47% of pregnancies with preexisting diabetes.^9^

While there is good evidence to show that PPC can significantly reduce adverse pregnancy outcomes,^10^–^13^ uptake of this care is limited, particularly in women with T2DM.^3^,^4^,^9^ Consequently, studies consistently showed that women with T2DM were generally more likely to be poorly prepared for pregnancy compared to those with T1DM.^3^,^4^,^14^ The NPID audit found that less than a third and a fifth of women with T2DM entering pregnancy care had adequately optimized glycemic control and been prescribed an appropriate dose of folic acid, respectively. They also reported that 10% of pregnant women with T2DM were still taking teratogenic drugs.^9^ It is also increasingly recognized that many pregnancies in women with T2DM are unplanned. Hence, PPC also needs to consider wider aspects of reproductive care, including the provision of effective contraception.

In the UK, a further impediment for accessing PPC in women with T2DM is that most T2DM care is delivered in primary care, whereas the majority of PPC is situated in specialist diabetes centers.^15^ Therefore, unlike T1DM, where the diabetes and PPC services are integrated, PPC is not co-located with routine T2DM care. This dissociation may impact on the accessibility of PPC to women with T2DM and restrict their access to health care professionals (HCPs) with expertise in reproductive health in the context of diabetes care.

To address this problem, interventions have been developed to enhance PPC uptake utilizing multimodal approaches that have included the following: patient education, health professional education, leafleting, and clinical guidelines.^3^,^4^,^14^ However, such interventions have shown limited increases in PPC uptake in women with T2DM. The reasons for this poor uptake are likely to be multifactorial, reflecting both the women’s beliefs about pregnancy risks and the limitations of current care delivery models (including the women’s interactions with HCPs). Therefore, to develop more targeted and effective interventions, it is important to better understand the barriers and facilitators involving a combination of person and system factors. To this end, we present a meta-synthesis of the views and experiences of women and HCPs on PPC.

## Methods

A systematic literature review with meta-synthesis was undertaken to address the following research questions:

1. What are women’s and HCPs’ views and experiences of PPC?
2. What factors contribute to the uptake of PPC?

### Synthesis approach

The approach adopted for this synthesis was meta- ethnography, following the methods outlined by Noblit and Hare^16^ as adapted for health services research by Britten et al.^17^ The synthesis was conducted in seven phases, and it is detailed as follows:

Phase 1: identify topic for synthesis: The review questions were developed from an analysis of current evidence for the uptake of PPC and an assessment of previously studied interventions to promote PPC uptake.

Phase 2: study identification: A systematic search was performed in the following databases: MEDLINE, Embase, PsycINFO, CINAHL, Maternity and Infant Care, and Web of Science. The search strategy was informed by an initial scoping review and included an extensive list of key terms and synonyms for the following: “type 2 diabetes”, “preconception”, “pre-pregnancy care”, and “pregnancy planning”. The titles and abstracts of all the identified studies were screened independently by two researchers and were selected according to the following inclusion criteria:

- Studies that addressed the topic of PPC
- Original research using qualitative methodologies
- Participants were women with T2DM or HCPs who care for them.

**Figure 1:**
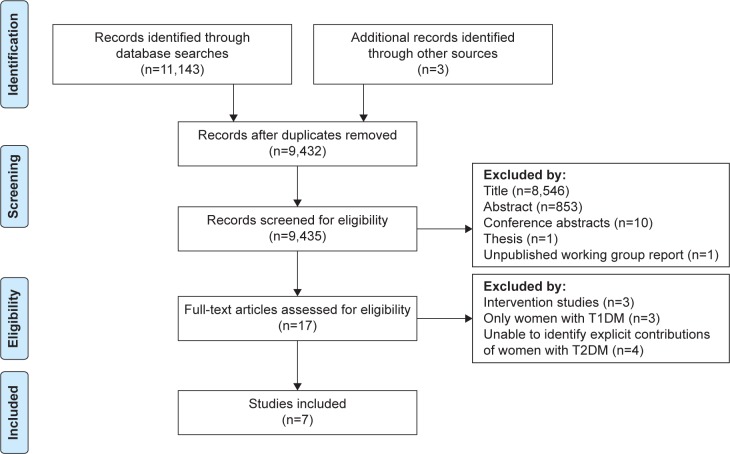
PRISMA flow diagram of database searches: summary of search strategy. **Abbreviations:** PRISMA, Preferred Reporting Items for Systematic Reviews and Meta-Analyses; T1DM, type 1 diabetes mellitus; T2DM, type 2 diabetes mellitus.

The interrater agreement between the two researchers was high with a Cohen’s kappa coefficient of 0.98. Given the complexity of the research topic, the electronic search was supplemented by secondary citation searching, key author searches and contacts with experts in the field of PPC.^18^ The results of the search are summarized in the Preferred Reporting Items for Systematic Reviews and Meta-Analyses (PRISMA) diagram in Figure 1.

Data were extracted from the identified studies using a data extraction tool detailing: the research question, the aims and design of the study, the theoretical approach and analysis methods, participant characteristics and setting, and the findings of the studies (themes and data extracts). All of the identified studies included women with both T1DM and T2DM; however, only the data attributed to women with T2DM were extracted for inclusion in the synthesis. The data from the women with T2DM and HCPs were handled independently as separate syntheses. The extracted data were organized into first- and second-order constructs. First-order constructs were all the verbatim quotes attributable to women with T2DM or HCPs in each study, and the second-order constructs were the themes identified in the studies. Each study was critically appraised and scored using the Critical Appraisal Skills Programme qualitative research appraisal tool.^19^ Data extraction and critical appraisal were undertaken independently by two researchers separately and then discussed until a consensus was reached.

Phase 3: initial reading and familiarizing: All of the articles were scrutinized thoroughly by two researchers; this involved reading and rereading each study.

Phase 4: determining how the studies are related: This involved a thematic tabulation of the primary data from all the studies to allow cross-study comparisons, integrate the themes from each study, and facilitate the identification of new concepts.^17^,^20^,^21^ This tabulation was independently verified by two researchers.

Phase 5: translating the studies into one another: The first- and second-order constructs from each study were viewed within the tabulation detailed in phase 4 to develop third-order constructs, which are new ways of considering the studied phenomena. This involved translating the first- and second-order constructs from the original studies within a comparative matrix from which integrated themes were identified. These integrated themes were then used to identify the third-order constructs.

Phase 6: synthesizing the translations: A line of argument synthesis was used to validate the emerging themes and third-order constructs using both reciprocal (where findings are concordant) and refutational (where findings are divergent) synthesis. This ensured that the new third-order constructs identified provided a novel assessment of the collected data, while remaining connected to the data from the original studies. This synthesis involved collective discussions by the two researchers who conducted the previous phases of the synthesis.

Phase 7: communicating the synthesis: This must provide an account that enhances an understanding of the observed phenomena in the context of the underlying purpose of the inquiry.^16^ A line-of-argument synthesis recognizes that studies address different aspects of a phenomenon.^22^ Therefore, to better understand what contributes to the uptake of PPC among women with T2DM, which is the phenomena under investigation, this synthesis explored the experiences of women with T2DM and HCPs. An integrated model was then developed formulating the relationships between the identified constructs which also highlight areas for further study.

## Findings

A total of seven studies met the inclusion criteria exploring the experiences and views of 28 women with T2DM and 83 HCPs from primary and secondary care inclusive of all members of the multidisciplinary team. Table 1 provides an overview of all the included studies, and Table 2 summarizes the second-order constructs supported by examples of first-order data from each study.

The authors identified four third-order constructs related to the synthesis of the data pertaining to women with T2DM and two constructs expressing the experiences of HCPs. A detailed description of each of these constructs and their subthemes are presented in the following sections, supported by data from the original studies.

### Construct 1: understanding PPC

The extent to which the women engaged with PPC was reflective of their understanding of the relationship between T2DM and pregnancy and the need for PPC.

#### Theme 1: information provision

Many women reported a lack of engagement with PPC, because they were not informed about why it was needed, and one woman stated that “she would have attended preconception care had she known that the service existed.”^23^ The information was often delivered in an ad hoc way, such that the women could conceive prior to the information being imparted.

I’ve only been told the second time I went [for clinic appointment], and they said “Oh, by the way, if you’re planning to get pregnant, you know, you have to go on insulin ….” I could have got pregnant all that time before!^24^

#### Theme 2: interactions with HCPs

Women with T2DM reported that the way HCPs approached a discussion about pregnancy and the need for PPC influenced how receptive they were to the guidance given.

It’s just the whole attitude, you know, it was like it didn’t matter … maybe I’m just one person, but it’s important for me if it’s not for them.^24^

Some women perceived that HCPs did not consider their reproductive needs or provide supportive information about pregnancy or PPC due to biased views about their age or because they were obese.

I feel they just look at me and say, “she’s fat – just push her off to one side and that’s it,” … that’s how I feel all the time.^24^

#### Theme 3: perceived relevance of PPC information

The women’s engagement with PPC was related to how receptive they were to receiving information on planning for pregnancy. Some women reported that they did not want information about PPC and were ambivalent about a potential pregnancy. Others avoided discussing plans for pregnancy with their health care team, as it might be a “deterrent to having children”.^24^

I mean we weren’t really thinking about it (pregnancy) at that time so I mean it was all like pushed to one side. Yeah, so I probably wouldn’t have taken much notice about it.^25^

The focus of the information and its perceived relevance also influenced how the women responded to PPC advice. Some women reported having received PPC but felt that the central focus was on general factors such as diet and exercise rather than the relationship between diabetes and a future pregnancy. The women felt that the relevance of information would be enhanced if it emphasized the potential benefits of PPC for both themselves and their offspring, as a partner of one woman voiced:

She didn’t like the horror stories as she calls them; all the bad things that could happen …. I suppose looking back, in hindsight, they could have counterbalanced that with all the good things that could happen. (Partner)^25^

### Construct 2: emotive catalysts

Many of the women voiced strong emotions (guilt, fear, and anxiety) about their reproductive capacity and risks. These emotional responses were associated with concerns for the health of a future baby and the loss of control over their reproductive intentions. These emotive catalysts can induce negative behavioral responses such as avoidance behaviors in respect of PPC.

#### Theme 1: health concerns for their offspring

The decision to become pregnant while having diabetes can lead to anxieties as to whether they would be able to “adequately control” their diabetes to protect their baby. Such thoughts may inhibit women from engaging in PPC if those anxieties were not addressed.

It’s more miscarriage, its more stillbirth … […] but it seems to me that if you’ve already got problems they just escalate, so I don’t know, I’m frightened … you just have to wait and see if something really horrible happens to it [baby].^23^

Previous pregnancy experiences can heighten women’s concerns about their offspring. One woman while reflecting on a previous unplanned pregnancy described how her experience would make her more likely to take up PPC for a future pregnancy.

I’ve never been this scared or shocked in my whole life … it’s still sinking in so I would definitely want to get my, the whole of my body sorted before I did anything like this ever again.^25^

#### Theme 2: pregnancy eclipsed by diabetes

Women also expressed emotions in relation to how their diabetes may lead to a loss of personal control over their pregnancy. Such perceptions were a deterrent to engaging with PPC as they wanted a normal pregnancy experience and PPC could mean that even prior to their pregnancy their diabetes would take precedence.

I would not want to bother with it’ cause I would want that bit of my life to be as normal as possible.^25^

The women also reported an underlying anxiety about their ability to control their diabetes and how this could impact on their pregnancy.

… this may sound really silly but by focusing on the diabetes, I feel I lose control of it because it nerves me, it frightens me then and I start panicking about it [pregnancy].^23^

### Construct 3: beliefs among women with T2DM about reproduction

Beliefs and cultural perspectives about reproductive health were strongly reflected in the women’s narratives. These beliefs were influential in the women’s reproductive behaviors, including internalized beliefs about reproduction and diabetes and the desire to conform to cultural norms.

#### Theme 1: beliefs about contraception

The women reported contrasting beliefs about contraception. There were beliefs that contraception was not necessary because the women’s diabetes meant they would not get pregnant or that contraception, particularly oral contraception, was inappropriate for people with diabetes.

Well my doctor didn’t give me the pill … No, “cause I’ve got diabetes and you know, I’m not the most em, the best diabetic I think …”^25^

Some beliefs may have resulted in the suboptimal use of contraception.

… I wasn’t takin’ it as regular as I should have done. Yeah, I was sort of missing one and thinking Oh that’s all right, I’ll take two in the morning and … it doesn’t work like that, does it?^25^

#### Theme 2: fertility beliefs

Beliefs about fertility and diabetes among women with T2DM influenced their behaviors related to pregnancy planning. In particular, some women believed that diabetes or the consequence of diabetes would diminish their fertility reducing the perceived relevance of PPC.

… because I didn’t ever think that I would be able to have children. I just thought that that was a lot harder to conceive with having diabetes.^25^

#### Theme 3: ambivalence toward planning pregnancy

Some women with T2DM held the belief that pregnancy outcomes were more down to chance or the “luck of the draw”.^24^ This belief undermined the significance of diabetes for pregnancy, and they did not recognize the importance of PPC. As one multiparous woman relayed: “[they were] all surprises … all just happened.”^26^

### Construct 4: relationships and social factors

External relationships particularly the views of partners were influential in women’s pregnancy planning behaviors. For some women, gender power dynamics were evident in these relationships with women ceding control to their partners over their reproductive practices.

Yes I was given information by my GP’s nurse, but I did not use contraception … My husband said, do not use any contraception we want a baby.^25^

Other women recounted that having a supportive relationship increased their readiness to plan for pregnancy.

… We finally sort of sat down and said, Look you know, if we’re going to do this, we need to do it now, or forget about it.^26^

### Construct 5: HCP behaviors and perspectives

The accounts of HCPs identified a number of underlying perspectives on PPC that influenced their behaviors in relation to the initiation of PPC and the advice and support they provided to women.

#### Theme 1: HCPs’ understanding of PPC

The extent to which HCPs engaged in PPC was related to their understanding of the needs of women with T2DM. Some HCPs felt this aspect of diabetes care was not adequately addressed in their professional education, and hence they were unaware and ill-prepared to deliver PPC.

Not much attention was paid to this issue during the training courses, and now that we have only received subtle training; our information is not sufficient.^27^

Knowledge deficits impacted on the proficiency of the care they provided, with some HCPs believing that care can be optimized after, rather than before conception. There was also a tendency to focus on glucose control, neglecting other aspects of PPC, such as discontinuing teratogenic medications.

… if they got pregnant and I would get there control as good as it was and I’d emphasize they must come and see us as soon as they’re pregnant.^28^

Some HCPs reported that they were unaware that women with T2DM need PPC, but if informed that they would be more mindful of providing this care.

I’ve never really thought about this topic before but … I would say it’s definitely in primary care, we have to be aware, we have to highlight these cases where we probably hadn’t been doing.^28^

#### Theme 2: recognizing reproductive potential

Many HCPs acknowledged that women with T2DM were not routinely considered in terms of their reproductive potential. Consequently, PPC was often not incorporated into diabetes care for these women.

… part of the problem is, type 2 diabetes for a lot of people is seen as a disease of the elderly. And more of our patients are middle-aged to old people, so thinking about pregnancy, is not automatic at all …^28^

Some HCPs recognized the importance of avoiding and actively planning for pregnancy, as one general practitioner (GP) suggested that many women miss out on appropriate reproductive care if the focus is exclusively on those who declare a pregnancy intention.

I actually think rather than talking about preconception care, you talk about the … care of the potentially pregnant, rather than preconception, because you’ll miss a lot of people because people won’t be ready for preconception care advice.^28^

#### Theme 3: HCPs’ perceptions of PPC

In general, HCPs perceived PPC to be very demanding for women and tended to emphasize the work commitments required and hazards in relation to pregnancy, rather than emphasizing the potential benefits of PPC.

The whole course of the pregnancy is going to be a lot of work. She’s going to be doing her blood sugars a lot more often than she usually does and her diet is going to have to be under much tighter control.^29^

Some HCPs also held skeptical views about women’s ability to adhere to the self-management practices involved in PPC and pregnancy.

I think it’s just denial … [they think] Nothing’s going to happen to the baby. I’ll be OK if I skip a few days of my medication. I’ll be Ok if I don’t check my sugars …^29^

#### Theme 4: the impact of sociocultural factors

HCPs identified that underlying sociocultural factors may influence how women view pregnancy. They attributed information deficits in relation to PPC to language and literacy deficiencies, particularly among women with T2DM from ethnic minority communities.

Language problems, education level and not being able to read, not comprehending.^29^

They also felt that cultural factors within families and communities influenced women’s reproductive behaviors to a greater extent than HCP advice.

It’s really interesting, the cultural things going on sometimes. In what he [the husband] perceives to be good for the pregnancy or what the patient’s mother-in-law thinks should go on for their future grandchild.^29^

Addressing the views and knowledge deficits of family members was identified by one HCP as being helpful. However, cultural beliefs could be modified through addressing knowledge deficits helping to promote the uptake of PPC.

Husbands are usually unaware [about the need for pre-pregnancy care]. When they find out about the complications, they accept and pursue the matter.^27^

### Construct 6: health care system factors

Many HCPs identified limitations in the health care system as having a significant impact on the delivery of PPC.

#### Theme 1: integrated working

The views expressed by HCPs suggested a number of difficulties with care integration that inhibited PPC. A particular challenge relates to the delivery of care across different services and professional disciplines. One area of complexity was the relationship between primary and specialist diabetes care. HCPs described a lack of shared understanding and agreement between these services in how to best provide PPC and who should provide the care. There was a perception among some specialist HCPs that primary care professionals lacked the skills and resources to deliver PPC.

I would be very happy for GPs to deliver preconception care and then to tap into our system, into our team […] The problem is that some GPs … have no interest at all and their general level of diabetes care is very poor.^28^

In contrast, other HCPs emphasized that it was not about who delivered the care, but rather the need to ensure the provision of reliable consistent information across the multidisciplinary team.

I don’t think it matters who gives the message as long as we’re all giving the same consistent message, GPs, practice nurses, health visitors, community midwives, everyone …^28^

HCPs offered ideas on how to enhance care integration suggesting a more blended and collaborative care approach.

You might do a bit of collaboration and mix and match … I might say why don’t you [the patient] go and have a one-off appointment, talk about what are issues for preconception care, maybe … and then let us [primary and secondary care] share the care.^28^

#### Theme 2: resource constraints

The HCP accounts suggested that a barrier to women accessing PPC was either implicit or explicit care rationing. There was a perception that referring women to a specialist PPC clinic may not be an appropriate use of resources, as expressed by this primary care HCP:

If they’re not pregnant when do you make the referral without hugely increasing the workload of the clinic at hospital? Will they be able to cope if you’re referring 10 women who are trying to get pregnant but none of them are pregnant.^28^

The resource question was further complicated by the impact of remuneration and commissioning models. These models can reduce the incentive for HCPs to refer women to PPC, as they may be outside of current remuneration frameworks particularly in primary care.

The government has set up conflicting incentives. On the one hand you must identify all disease groups and all risks. And be judged upon how well you do that. On the other hand if you refer patient to hospital, you’re going to be financially penalized.^28^

#### Theme 3: system-level constraints

For many HCPs, particularly those in primary care, rationing in the organization and delivery of health care placed significant constraints on the PPC they provided. The most significant of these was the difficulty in incorporating a PPC strategy within an overburdened care system.

[…] you’re under such pressure in terms of time in a general consultation, you know, you might have 10 minutes, it’s um, unless there is a system that alerts you to consider it, it’s unlikely to get into, you know, you’re just dealing with now.^28^

HCPs also identified that the overall care system is orientated to priorities other than PPC. In primary care, priority is given to clinical outcome targets and compliance with following embedded protocols that do not include PPC.

People often go by what’s on the templates and, you know, that’s not a part of it. So, I think if you’re rushing and you’re filling in templates, it’s not on it, it might be an idea to put that in …^28^

## Integrative model

**Figure 2:**
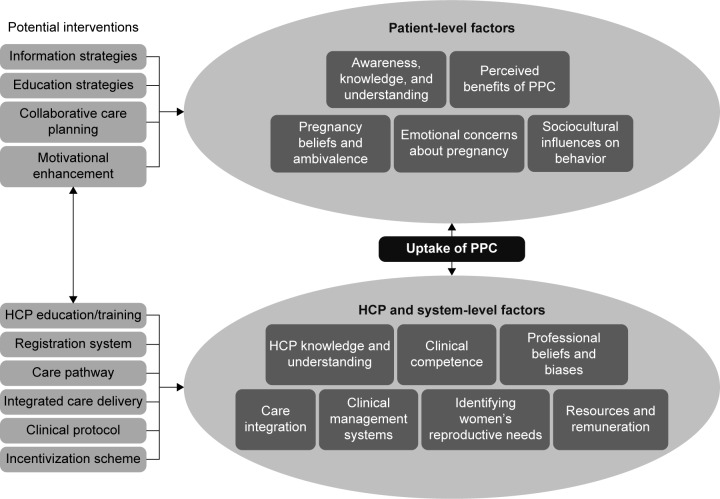
Integrated model of elements contributing to PPC uptake and potential interventions. **Abbreviations:** HCP, health care professional; PPC, prepregnancy care.

To represent the co-dependent relationships between the patient, HCP, and system-level elements contributing to the uptake of PPC, a model is shown in Figure 2. This illustrates the complex nature of the issues involved in providing support for women with T2DM in managing their reproductive needs. At the patient level, this extends beyond having an awareness of why it is important to manage fertility or prepare for pregnancies, to include an understanding of the emotional, cognitive, motivational, and social phenomena that influence their health-seeking behaviors and responsiveness to care. Similar factors contribute to the care-providing behaviors of HCPs and indeed their awareness, knowledge, clinical skills, and beliefs about the reproductive needs of women with T2DM. The model also highlights the multiple care system limitations that prejudice the delivery of appropriate PPC. In presenting competing priorities and tensions within the wider care system, it was possible to identify key areas for intervention to enhance PPC uptake. The limitations in time and specialist resources combined with malaligned financial incentives prejudice the delivery of appropriate PPC, and these areas are explored further in the “Conclusion” section.

## Conclusion

This synthesis, generated from the integrated views and experiences of women with T2DM and HCPs, has provided a deeper understanding of elements contributing to the uptake of PPC among women with T2DM. It is clear that the uptake and effectiveness of PPC require more than simply providing information to women about the importance of managing their reproduction and pregnancy planning.^30^ While such information may increase the awareness of women about PPC, underlying beliefs, emotional concerns, and social factors also need to be considered to ensure that the women become actively engaged with and benefit from PPC. This suggests that in addition to using various media for a multimodal and staged approach, women’s beliefs and anxieties about pregnancy are actively explored prior to interventional strategies to increase the women’s engagement and activation in relation to their reproductive health. This may require the development of more collaborative and motivational methods of care delivery such as collaborative care planning.^5^

The synthesis has highlighted that the approach of HCP to woman in providing support is also important. Previous studies have emphasized that a positive relationship between the patients and HCPs can influence the experience of PPC for women with T1DM.^31^ However, this synthesis has shown that HCPs often do not consider the reproductive potential of women with T2DM and in some cases may hold negative views of the women, particularly in relation to weight, prejudicing discussions about pregnancy.^24^ Hence, HCPs should be supported in developing consultation skills that elicit women’s pregnancy intentions, allow them to voice any concerns or anxieties, and foster a productive relationship for the delivery of PPC.^32^,^33^

The synthesis of both the women with T2DM and HCPs identified the impact of social and cultural factors on women’s reproductive choices. Interventions may need to consider not only the women’s views but wider family and community influences. Social relationships, especially spouses, may be influential, involving them in the PPC discussion may help women adopt protective behaviors by encouraging women to voice their beliefs and views on pregnancy. At a wider level, it may be useful to work with specific ethnic communities to help promote an understanding of PPC. Given the preponderance of diabetes in women of childbearing age in some ethnic communities, such approaches may enhance PPC awareness and uptake.^34^

Patient education has a role to play in promoting an understanding of reproductive health issues in diabetes. Women observed that there can be a delay between diagnosis of diabetes and when they receive advice about pregnancy. During that period, women are vulnerable to an unplanned pregnancy. Currently, while all people newly diagnosed with T2DM are offered structured education,^35^ most education does not address reproduction.^36^ Therefore, education strategies may be useful soon after diagnosis and need to address the wider factors associated with women’s reproductive behaviors. Women reported that experiencing a pregnancy can influence future pregnancy planning, thus it may be useful to include the personal experiences of multiparous women as part of educational resources.

The HCPs’ accounts suggest that there is a need to extend training and skills in managing the reproductive care needs of women with T2DM, especially in primary care where most T2DM is managed. Such training needs to consider the requirements of women not only actively aiming for pregnancy but also preventing unplanned pregnancies. HCP education needs to address potentially unhelpful beliefs about obesity, age, and diabetes in terms of fertility and fecundity. Education should challenge HCP biases that can negatively impact the therapeutic relationship with women with T2DM.

Finally, unlike the general population where ∼55% of pregnancies are considered planned,^37^ women with T2DM require specific care to target their additional risk factors. This synthesis has identified that the current care system is not orientated to promoting and delivering effective PPC for women with T2DM. There are inconsistencies in the care provided and lack of integration between primary care and the specialist diabetes teams running PPC services. While the recent guidelines for pregnancy in diabetes^5^ incorporating PPC and current practice make many recommendations, there is a disconnect between PPC models of delivery; hence, new strategies are required to implement these guidelines. PPC has been more prudently addressed for other medical conditions, such as epilepsy, and the resulting rates of planned pregnancies are more reflective of the general population.^38^ Internationally, there are registries for epilepsy and pregnancy,^39^,^40^ and within the UK the provision of PPC for these women is incentivized within primary care.^41^,^42^ Future recommendations from our findings suggest that a new model of service delivery is required for women with T2DM. This new approach would incorporate comprehensive methods for registering and reviewing women of childbearing age, better care protocols with built-in decision-making tools and prompts, and clear clinical pathways with criteria for referral to specialist PPC. In the UK, a significant barrier is that reproductive health in diabetes is not part of the national incentive program in primary care and consequently is given a low priority.^43^ Incentivizing PPC or bringing in performance monitoring either locally or nationally may increase the performance in reproductive care.^44^ While some areas of the UK have already begun to adapt systems to enhance the uptake of PPC,^45^,^46^ these developments have not targeted women with T2DM specifically and the impact has not been systematically evaluated.

### Limitations

While the review was designed and conducted following an explicit and rigorous methodological approach, there are some limitations to consider. Principal among these were the limited number of qualitative studies for the synthesis and the fact that these studies were not exclusive to women with T2DM. While we attended to the latter issue by only including data from women with T2DM, this restricted the scope of our interpretation and hence there may be additional factors that we have not identified. Nevertheless, the synthesis has provided novel insights into the collective factors impacting on PPC. These insights will be a useful reference for further exploratory studies and in developing new interventions.

In conclusion, the limited uptake of PPC is multifactorial and depends on patient, HCP, and care system. The findings suggest that multimodal approaches are required to improve PPC uptake with a view to optimizing the health of women with T2DM and their babies and reducing the number of unplanned pregnancies. Acknowledging the complexities exposed in this synthesis, the development of such approaches should involve women with T2DM and HCPs to ensure that interventions are sensitive to their needs. The synthesis also emphasizes the need to address system- and policy-level inconsistencies.

## Figures and Tables

**Table 1:** Included studies for meta-synthesis

Study	Country	Aim	Participant characteristics	Data collection	Data analysis	Themes (second-order constructs)
Spence et al^24^	Northern Ireland	To determine the knowledge and attitudes of women with T1DM and T2DM of childbearing age toward PPC	18 women with T1DM Six women with T2DM: all Caucasian Six women with T2DM 2/6 Caucasian 4/6 Asian	Convenient sample Focus group interviews	Content analysis	Knowledge Quality of relationships with HCPs Organization of care The impact of beliefs and attitudes on advice giving Women’s attitudes to PPC advice
Murphy et al^25^	UK	To explore the views of women with diabetes who did not attend PPC	21 women with T1DM Eight women with T2DM 76% Caucasian and 21% Asian/Pakistani	Semi-structured interviews	Thematic analysis	Women’s views of preconception counseling Contraceptive behaviors Knowledge regarding the risks of diabetes during pregnancy Past pregnancy experiences Becoming pregnant Attending PPC
Lavender et al^23^	UK	To explore the experiences of White British and South East Asian women with T1DM and T2DM and the perceived impact of diabetes on their reproductive health	15 women with T1DM Seven women with T2DM Seven women with T2DM 4/7 South East Asian 2/7 Caucasian 1/7 Black African	Purposive sample	Hermeneutic phenomenological approach	Relinquishing personal control Pregnancy overshadowed by DM Haphazard PPC
Letherby et al^26^	UK	To explore the experience of women with pregnancies complicated by diabetes	Eight women with T1DM Four women with T2DM Ethnicity not specified	In-depth interviews	Grounded theory	Timing of pregnancy
Nekuei et al^27^	Iran	To gain insight into experiences of diabetic women and providers about PPC	Five women with T1DM Three women with T2DM 15 HCPs	Purposive sampling Semi-structured individual interviews	Conventional content analysis	Health centers’ weakness in providing PPC for diabetic women Lack of a comprehensive PPC plan for diabetic women Diabetic women’s negligence about having planned pregnancy
Mersereau et al^29^	USA	To explore the knowledge, attitudes and barriers that pregnant women with DM and their practitioners face with respect to achieving good glycemic control to reduce adverse effects for women and infants	53 HCPs consisting of physicians, midlevel practitioners, and certified diabetes educators	Convenient sample Focus group interviews	Thematic analysis	HCPs consider most pregnancies are unplanned HCPs focus on the health and well-being of the baby Women express concerns for the baby rather than themselves Barriers to care are a lack of knowledge, lack of access, and attitude to the need for care
Mortagy et al^28^	UK	To examine the perspectives of GPs and secondary-care HCPs on current and envisaged roles and responsibilities of GPs in delivering PPC to women with DM	Eight GPs One endocrinologist One obstetrician Three diabetes nurses One diabetes midwife One dietitian	Semi-structured interviews	Thematic analysis	Case load and patient profile Ambiguity of GP roles and responsibilities Missed opportunities Integration of care

**Abbreviations:** DM, diabetes mellitus; GPs, general practitioners; HCPs, health care professionals; PPC, prepregnancy care; T1DM, type 1 DM; T2DM, type 2 DM.

**Table 2:** First- and second-order constructs

Study	First-order constructs (sample of quotations)	Second-order constructs (themes in articles)
Spence et al^24^	“I’ve only been told the second time I went [for clinic appointment], and they said ‘Oh, by the way, if you’re planning to get pregnant, you know, you have to go on insulin’. So I thought that’s … I could have got pregnant all that time before!” “It’s just the whole attitude, you know, it was like it didn’t matter … maybe I’m just one person, but it’s important for me if it’s not for them.”	Knowledge Quality of relationships with HCPs Organization of care The impact of beliefs and attitudes on advice giving Women’s attitudes to prepregnancy care advice
Murphy et al^25^	“I mean we weren’t really thinking about it at that time so I mean it was all like pushed to one side. Yeah, so I probably wouldn’t have taken much notice about it if they would have said anything.” “Yes I was given information by my GP’s nurse, but I did not use contraception … My husband said, do not use any contraception we want a baby.” “I went on to insulin and I was on insulin for a year or two trying to have a baby and they said they didn’t think I could … then I changed doctors last year because mine was useless and they put me on a tablet [Rosiglitazone] and 7 years later I’m pregnant …”	Women’s views of preconception counseling Contraceptive behaviors Knowledge regarding the risks of diabetes during pregnancy Past pregnancy experiences Becoming pregnant Attending PPC
Lavender et al^23^	“She would have attended preconception care had she known that the service existed.” “It’s more miscarriage, its more stillbirth. I mean, I know that doesn’t always have to be the way but it seems to me that if you’ve already got problems anyway they just escalate and escalate, so I don’t know, I’m frightened … you just have to wait and see if something really horrible happens to it [baby] but you just do your best to try and make sure that it doesn’t.”	Relinquishing personal control Pregnancy overshadowed by DM Haphazard PPC
Letherby et al^26^	“… from a time perspective that, one – being diabetic and secondly knowing that I was getting older … we finally sort of sat down and said, Look you know, if we’re going to do this, we need to do it new, or forget about it.”	Timing of pregnancy
Nekuei et al^27^	“Not much attention was paid to this issue during the training courses, and now that we have only received subtle training; our information is not sufficient.” “Preconception consultation requires a great deal of time as well as skill. Responsible personnel for this practice do not have the needed motivation to spend their time on this issue.” “All members of the health team shift the blame onto each other and think that they have done their duties correctly.”	Health centers’ weakness in providing PPC for diabetic women Lack of a comprehensive PPC plan for diabetic women Diabetic women’s negligence about having planned pregnancy
Mersereau et al^29^	“They’re more concerned about are they going to have to take insulin … they don’t want to check their sugars.” “The type 2’s especially … haven’t been educated on the importance of getting their blood sugars in control prior to getting pregnant and getting prenatal care started.” “It’s really interesting, the cultural things going on sometimes. In what he [the husband] perceives to be good for the pregnancy or what the patient’s mother-in-law thinks should go on for their future grandchild.”	HCPs consider most pregnancies are unplanned Women express concerns for the baby rather than themselves Barriers to care are a lack of knowledge, lack of access, and attitude to the need for care
Mortagy et al^28^	“… part of the problem is, type 2 diabetes for a lot of people is seen as a disease of the elderly. And more of our patients are middle-aged to old people, so I think the kind of, you know, diabetes, think about pregnancy, is not automatic at all …” “Frankly [in] general practice, you’re under such pressure in terms of time in a general consultation, you know, you might have 10 minutes, it’s um, unless there is a system that alerts you to um, to consider it, it’s unlikely to get into, you know, you’re just dealing with now.”	Case load and patient profile Ambiguity of roles and responsibilities of GPs Missed opportunities Integration of care

**Abbreviations:** DM, diabetes mellitus; GPs, general practitioners; HCPs, health care professionals; PPC, prepregnancy care.
